# Clozapine Pathway Navigator: A Social-Work–Led Service to Accelerate Initiation and Improve Retention in Treatment-Resistant Psychosis

**DOI:** 10.1192/j.eurpsy.2026.12194

**Published:** 2026-07-31

**Authors:** M. Routsi, P. Argitis, M. Demetriou, C. Tsamis, S. Moschat, S. Bellos, Social

**Affiliations:** 1 Psychiatric; 2 General Hospital of Corfu, Corfu; 3 Psychiatric, General Hospital of Filiates, Igoumenitsa; 4 Psychiatric, University Hospital of Ioannina, Ioannina, Greece

## Abstract

**Introduction:**

Clozapine is under-utilised due to social and organisational barriers that delay initiation and compromise retention. A social-work “navigator” may address practical frictions, improve monitoring adherence and support continuity of care.

**Objectives:**

Determine whether a navigator shortens time from confirmed eligibility to clozapine initiation. Secondary: 3- and 6-month treatment retention, adherence to absolute neutrophil count (ANC) monitoring, motor adverse effects and health-related quality of life (QoL). and unplanned emergency utilisation and equity of access.

**Methods:**

Single-centre, before–after service evaluation in a specialised severe mental illness clinic. Adults with treatment-resistant schizophrenia-spectrum disorders meeting guideline criteria were included. The navigator delivered shared decision-making/psychoeducation; coordinated baseline work-up (CBC/ANC, ECG, metabolic panel, CRP and high-sensitivity troponin during titration per local protocol); arranged reminder/recall for ANC; provided benefits/housing/employment/transport support; and liaised with prescribers and primary care. Consecutive pre-implementation usual-care cases served as comparator. Outcomes: time-to-initiation (days); retention (on treatment at 3/6 months); ANC adherence (on-schedule draws, % per current EMA label/local policy); motor adverse effects (Simpson–Angus Scale; Abnormal Involuntary Movement Scale); QoL (EQ-5D-5L index/VAS); emergency department (ED) visits/bed-days. Analysis: Kaplan–Meier and Cox models for time-to-initiation; logistic regression for retention and ANC adherence; Poisson/negative binomial for utilisation; interrupted time-series sensitivity analyses with prespecified covariates.

**Results:**

Data from n=148 (pre=74, post=74). Median time-to-initiation decreased from 90 to 58 days; Cox HR 1.47 (95% CI 1.09–2.00, p=0.01). Three-month retention increased from 60% (44/74) to 78% (58/74) (adjusted OR 2.25, 95% CI 1.20–4.25, p=0.011); six-month retention 52% to 70% (adjusted OR 2.15, 95% CI 1.13–4.08, p=0.019). On-schedule ANC monitoring improved from 70% to 88% (adjusted OR 2.95, 95% CI 1.70–5.20, p<0.001). Motor adverse-effect changes were small (SAS Δ −0.2, 95% CI −0.6 to 0.2; AIMS Δ +0.1, −0.2 to 0.4). QoL improved (EQ-5D-5L index +0.06, 95% CI 0.01–0.10, p=0.02). ED visits fell from 1.0 to 0.7 per 100 patient-months (IRR 0.72, 95% CI 0.55–0.95, p=0.02). Sensitivity analyses were consistent.

**Conclusions:**

A social-work–led navigator is a pragmatic, scalable strategy to activate clozapine pathways. In this service, initiation was faster, monitoring adherence and short-term retention improved, and unscheduled utilisation decreased without a signal for increased motor adverse effects. Findings support scale-up and multisite evaluation with formal cost-effectiveness analysis.

**Disclosure of Interest:**

None Declared

